# Lost in Space? Unmasking the T Cell Reaction to Simulated Space Stressors

**DOI:** 10.3390/ijms242316943

**Published:** 2023-11-29

**Authors:** Silvana Miranda, Randy Vermeesen, Wilhelmina E. Radstake, Alessio Parisi, Anna Ivanova, Sarah Baatout, Kevin Tabury, Bjorn Baselet

**Affiliations:** 1 Radiobiology Unit, Belgian Nuclear Research Centre SCK CEN, 2400 Mol, Belgium; sfdsmira@sckcen.be (S.M.);; 2 Department of Biotechnology, Faculty of Bioscience Engineering, Ghent University, 9000 Ghent, Belgium; 3 Radiation Protection Dosimetry and Calibration Expert Group, Belgian Nuclear Research Centre (SCK CEN), 2400 Mol, Belgium; 4 Data Science Institute (DSI), I-BioStat University of Hasselt, 3590 Hasselt, Belgium; 5 Department of Biomedical Engineering, College of Engineering and Computing, University of South Carolina, Columbia, SC 29208, USA

**Keywords:** stress immunity, space radiobiology, altered gravity

## Abstract

The space environment will expose astronauts to stressors like ionizing radiation, altered gravity fields and elevated cortisol levels, which pose a health risk. Understanding how the interplay between these stressors changes T cells’ response is important to better characterize space-related immune dysfunction. We have exposed stimulated Jurkat cells to simulated space stressors (1 Gy, carbon ions/1 Gy photons, 1 µM hydrocortisone (HC), Mars, moon, and microgravity) in a single or combined manner. Pro-inflammatory cytokine IL-2 was measured in the supernatant of Jurkat cells and at the mRNA level. Results show that alone, HC, Mars gravity and microgravity significantly decrease IL-2 presence in the supernatant. 1 Gy carbon ion irradiation showed a smaller impact on IL-2 levels than photon irradiation. Combining exposure to different simulated space stressors seems to have less immunosuppressive effects. Gene expression was less impacted at the time-point collected. These findings showcase a complex T cell response to different conditions and suggest the importance of elevated cortisol levels in the context of space flight, also highlighting the need to use simulated partial gravity technologies to better understand the immune system’s response to the space environment.

## 1. Introduction

**Figure 1:**
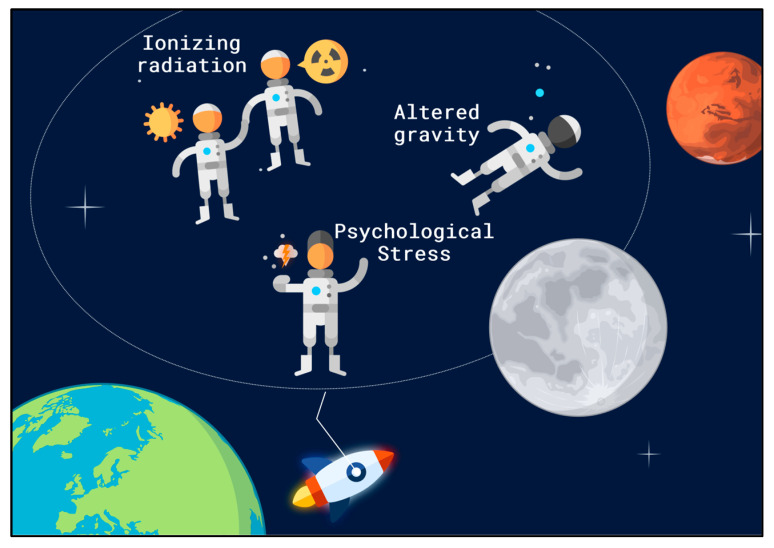
Space environment hazards astronauts will face in future space missions. Created with BioRender, icons by Freepik.

Space explorers are subject to an extreme hostile environment when they travel in outer space. This hostile environment poses numerous challenges to the health and well-being of astronauts in the form of physical challenges, such as increased ionizing radiation exposure and altered gravity [1] (Figure 1). Moreover, the nature and complexity of space missions also subject astronauts to significant psychological stress as a result of, amongst others, confinement, sleep deprivation, and a strenuous workload [2].

As we advance in the efforts for deep space exploration missions, it is important to also increase the effort for research on the effects of long-duration spaceflight on the human body and develop countermeasures to mitigate these risks.

### 1.1. Space Radiation

The ionizing radiation environment in space is multifaceted, with distinct characteristics in Low Earth Orbit (LEO), on the surface of other planetary bodies such as the moon, and in open space. It stems from a variety of sources, including galactic cosmic rays (GCRs) and solar particle events (SPE) that affect both LEO and open space. Furthermore, albedo particles are generated via the scattering of primary radiation by spacecraft structures or the interaction of GCRs and SPE with the lunar soil [3,4,5].

The majority (approximately 85%) of GCRs are composed of protons, with helium making up around 14%, and heavier particles contributing to approximately 1% of their composition. These high-energy heavy ions can penetrate spacecraft shielding, posing a significant risk to astronauts’ health due to their high linear energy transfer (LET) [4,6]. SPEs are events in which disturbances on the sun result in the release of intense bursts of ionizing radiation, primarily consisting of high-energy protons. These solar disturbances pose a significant concern for astronauts, as they can lead to severe adverse health effects, and they are of particular importance in the context of extravehicular activities (EVAs) [7]. The Van Allen Belts are regions within the Earth’s magnetic field where charged particles, including protons (inner belt) and electrons (inner and outer belt), become trapped along magnetic field lines that extend from pole to pole. These energetic particles can present a potential hazard to both space vessels and astronauts on future deep space exploration missions [3].

The effects of space radiation on human health are a matter of great concern for long-duration space missions; therefore, investigating the effects of space radiation is crucial for understanding the potential health risks faced by astronauts on deep space missions. However, access to samples from astronauts or the possibility of using real space platforms for these investigations is limited. There is a clear need to use ground-based facilities and technologies to simulate the space radiation environment and study its effects on biological systems [8].

Simulating space radiation is a crucial aspect of studying its effects on biological systems. Various methodologies and approaches have been developed to recreate the conditions of space radiation in laboratory settings such as particle accelerators which can be used to generate high-energy ions that mimic the composition of galactic cosmic rays and solar particle events. These particle accelerators allow researchers to expose biological samples to controlled doses of radiation and study the resulting effects [7,9].

### 1.2. Altered Gravity

In addition to the physiological changes induced by space radiation, altered gravity can also have profound effects on biological systems. Astronauts have been found to experience muscle atrophy, bone loss, cardiovascular deconditioning, and immune system dysfunction during prolonged space missions [10,11,12].

For the foreseen long-duration space flight missions to the moon or Mars, crew members will be exposed to different levels of gravitational pull. Although the detrimental effects of microgravity have already been under decades of investigation, there is a relevant knowledge gap relative to the effects of the partial levels of the moon and Martian gravitational fields [13]. The use of simulation technologies to expose in vitro models to altered gravity conditions can help bridge this knowledge gap and provide valuable insights into the potential biological effects of different gravitational forces. One common method is the use of random positioning machines (RPMs) which are devices that simulate altered gravity by randomly repositioning samples in three dimension in order to reduce orientation cues from gravitational force. Recent versions of RPMs allow for the simulation of the partial gravitational pull of the moon and Mars to study the effects of different gravitational fields on biological systems [14,15].

Ground-based experimental studies have shown that simulated microgravity can significantly impact cellular behavior, including changes in cell shape, gene expression, and cellular signaling pathways, in a similar manner to what is observed in space [16].

### 1.3. Psychological Stress

One of the major concerns for astronauts on long-duration space missions is the impact of stress on their physiological and psychological well-being. Stress is a complex physiological and psychological response to external or internal stimuli, often referred to as stressors. These stressors can include factors such as isolation, confinement, circadian rhythm and sleep disruption, altered gravity, and the immense mental pressure associated with the mission itself [17]. The stress response in humans is mediated by the hypothalamic–pituitary–adrenal axis, which leads to the release of cortisol, commonly known as the stress hormone [18]. Traveling deeper into the solar system implies that the distance from earth-based support systems increases. Crew members must be more self-reliant and are expected to perform independently. Furthermore, the living space area during a 21-month round trip to Mars is significantly smaller than the current size of the International Space Station (ISS), where most long-duration missions and studies have been conducted so far. It is expected that, in these conditions, the overall psychological stress experienced by the crew will be higher than what has been previously reported, such as increased cortisol levels demonstrated in astronauts [19,20,21].

The implications of prolonged elevated cortisol levels have yet to be elucidated in the context of space flight. Of particular concern are the effects on the immune system, since the effects of cortisol in chronic stress pathologies have already been shown to influence immune system function, such as a T cell shift from type 1 T helper (Th1) to Th2 cytokine profiles [22,23].

### 1.4. T Cells—Key Players in Immune System Function

The immune system is incredibly complex, and a plethora of different biomolecules, cells, and tissues work together to protect against threats such as microbes, viruses, cancer cells, and toxins. In this context, T cells are vital, as they help the activity of other immune cells, thereby polarizing immune responses into the appropriate type, depending on the nature of the immunological insult (virus vs. extracellular bacteria) [24]. In the context of T cell activation, interleukine-2 (IL-2) is an important cytokine for the recruitment of immune cells and maintenance of the immune response at the infection site. IL-2 is produced by activated T cells and plays a crucial role in promoting T cell proliferation as well as the differentiation of T cells into effector cells. Furthermore, IL-2 is involved in the development and function of regulatory T cells, which play a critical role in maintaining immune tolerance and preventing autoimmune diseases. The activation and function of T cells, including the production of IL-2, are essential for a robust immune response [25,26].

**Figure 2:**
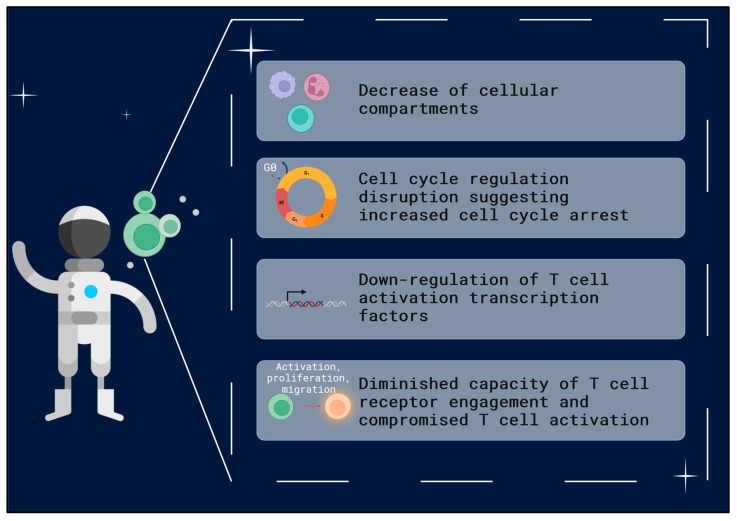
Main alterations described on T cells in the context of space environment exposure. Decrease in cellular compartments [29]. Cell cycle disruption [30]. Down-regulation of T cell activation [31]. Diminished T cell receptor engagement [32,33,34]. Created with BioRender, icons by Freepik.

An extreme environment, such as that found in space, offers a significant danger to the maintenance of T cell function [27]. Extensive work developed in past years has clearly shown a distinct immune dysfunction caused by space environment exposure [28]. When exposed to the space environment, several alterations were observed in T cells, such as a significant decrease in cellular compartments, cellular proliferation levels, and suppression of immune regulatory genes [29]. The disruption of proteins related to cell cycle regulation, with an increase in transcription of the cell cycle arrest protein p21, has been described [30]. In addition to cell cycle arrest, there is also evidence of down-regulation of transcription factors responsible for T cell activation [31]. Corroborating this negative impact of microgravity on the proliferation and activation of T cells, findings also show increased phosphorylation of the MAPK pathways, which corresponds to a diminished capacity for T cell receptors to be engaged, as well as other important signaling pathways crucial for cell function [32,33]. Moreover, gene expression studies have shown that there is a significant decrease in the expression of genes active in the early stages of T cell activation [34] (Figure 2).

Currently, the mechanisms underlying these alterations are unknown, and the potential interplay between the three primary space stressors (ionizing radiation, altered gravity, and increased stress) has not yet been elucidated.

Jurkat cells, a human T cell line, have been widely used in space research to understand the impact of microgravity on immune cell function. Previous studies have demonstrated that immune cell function is severely suppressed in Jurkat cells in microgravity [35].

**Figure 3:**
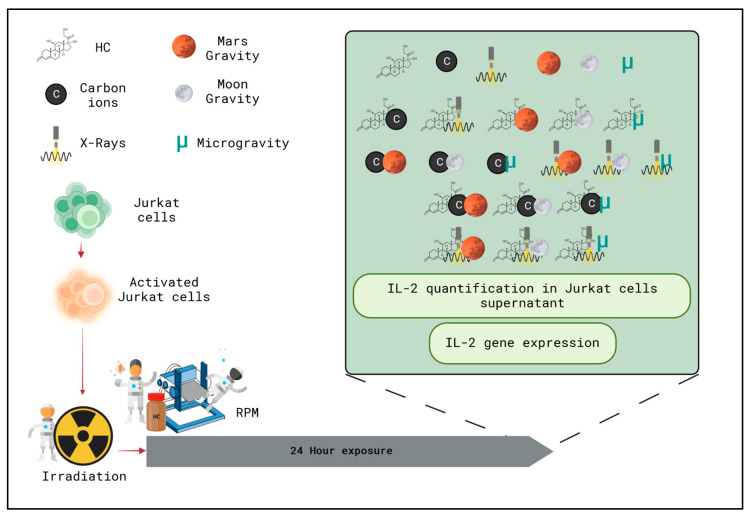
Schematic overview of the experimental design with the different experimental conditions of the simulated space stressors in a single and combined manner (green box). HC—hydrocortisone. Created with BioRender, icons by Freepik.

The goal of this manuscript was to investigate T cell activity after exposure to a combination of simulated space conditions (radiation with carbon ions and X-rays (photons), altered gravity (microgravity (Micro), moon gravity and Mars gravity), and stress with hydrocortisone (HC)) (see Table 1 and Figure 3 for reference). More specifically, T cell activity was evaluated by quantifying IL-2 expression at the mRNA and protein level in the supernatant.

## 2. Results

### 2.1. IL-2 Levels in the Supernatant of Jurkat Cells after 24 h Exposure to Simulated Space Conditions

To evaluate the secreted protein level of IL2 after 24 h exposure to simulated space stressors (single, double, and triple), the supernatant was collected and used for enzyme-linked immunosorbent assay (*ELISA*). Results were obtained from four different experiments where samples were divided into groups for each condition, with the control per condition sampled for every experiment. The IL-2 concentration was measured and normalized to the control group for every experiment.

#### 2.1.1. Single Simulated Space Stressor Effect

**Figure 4:**
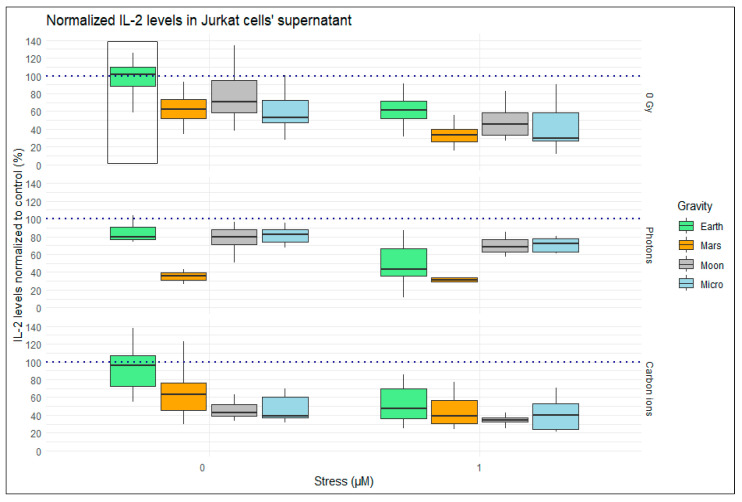
IL-2 measured on Jurkat cells’ supernatant. Measured with ELISA for IL-2 assay and processed with Belysa software (Merck). Values represented are normalized to the control (0 Gy, 0 µM stress, Earth gravity). Plot shows boxplot with median as center line. The bottom and top edges of the box represent the 1st quartile (Q1) and the 3rd quartile (Q3) of the data, respectively. The height of the box (the interquartile range or IQR) represents the middle 50% of the data. The whiskers represent 1.5 times the IQR. The data are divided in different panels based on the addition of stress hormone (stress (µM)) variable comparing the distribution of the normalized IL-2 values across different ionizing radiation exposures (0 Gy, photons, carbon ions). Gravity exposure is represented by different fill colors of the boxes (light green for normal “Earth” gravity, orange for simulated Mars gravity, gray for simulated moon gravity, and light blue for simulated microgravity (“Micro”)). The black line box highlights the control condition, and the horizontal dashed line indicates the average value for the control condition. There are 10–35 replicates per condition. Data analyzed with RStudio v4.3.1”.

We can observe that the addition of stress decreases IL-2 levels for every condition investigated, independently of the addition of other factors (Figure 4, right column—stress (µM) = 1). We also observed that the single exposure to Mars gravity and microgravity seems to have the most impact on the IL-2 secretion by the Jurkat cells. For normal (Earth) gravity levels, the two radiation qualities explored in our setup have less impact on IL-2 levels than HC exposure or simulated altered gravity. We observe that most values for photon irradiation at Earth gravity are below the average of the control and carbon ion irradiation groups. All the simulated gravity levels investigated show an impact on IL-2 levels. Interestingly, Mars gravity (~0.38 g) shows a higher negative influence on the IL-2 value than moon gravity (~0.17 g), indicating that the response might not be linear to the gravity vector value.

#### 2.1.2. Double Simulated Space Stressor Effects

Our experimental setup aimed to expose the cells to combinations of two of the simulated space stressors. We observed that the combination of stress and gravity levels further reduces IL-2 levels when compared to the use of gravity alone. The same is observed when we combine radiation and the different partial gravity levels. For the carbon ions’ irradiation, we can observe a decrease that is linear with the gravity vector value: a lower IL-2 value with lower gravity levels. This relationship is not observed for the photon irradiation, where we again see less IL-2 when combining 1 Gy photons and Mars gravity.

#### 2.1.3. Triple Simulated Space Stressor Effects

When we observe the results of IL-2 levels for the conditions that have a combination of three factors (Figure 4, 1 µM stress, altered gravity boxes in photons and carbon ions rows), we observe the impact that stress has on IL-2 levels. For all the combinations, 1 µM of HC reduces IL-2 levels further, compared to the conditions where we only have radiation and altered gravity exposure.

A linear regression model was employed to investigate the combined influence of the type of radiation, the addition of stress, and exposure to altered gravity, on IL-2 levels or gene expression. This approach allowed us to explore the main effects of these factors as well as their potential interactions. The data were analyzed using robust analysis to account for the lack of normality of the residuals of the linear model observed (see Figure S1) [36,37]. Outliers observed were removed from the dataset prior to analysis.

**Figure 5:**
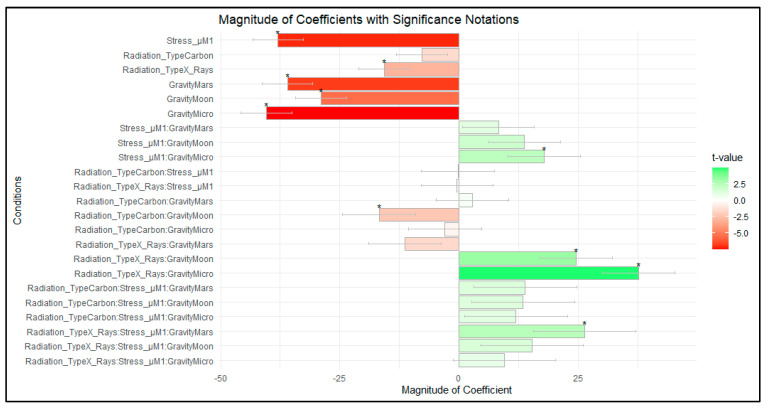
*t*-value ranges and the relationship between coefficient magnitude and significance. A robust linear model was applied to analyze the weighted data of the IL-2 values after exposure to simulated space conditions. In the bar plot, *t*-values represent the significance of the coefficients for different conditions, while the coefficients’ magnitude indicates the size of the effect each condition has on the response variable. A *t*-value greater than 1.96 or less than −1.96 is considered statistically significant (marked with *). The color gradient, from red to green, illustrates both the magnitude and significance of the *t*-values. Red indicates negative *t*-values, reflecting a negative impact, while green indicates positive *t*-values, showing a positive impact. The darker the shade, the larger the magnitude of the *t*-value, emphasizing the strength of the effect. Data analyzed with RStudio v4.31.

Since the experimental data were obtained across a series of experiments, we recognized the importance of accounting for variations in sample size when evaluating the relationships between IL-2 levels and various factors, such as radiation type, stress levels, and gravitational conditions. To address this, we initially calculated the sample size for each combination of these factors in our dataset. These sample sizes were then used to derive weights, assigning higher weights to observations from smaller sample sizes to ensure that all data points were equally considered and ensure that the analysis was not dominated by larger groups. A robust linear model (rlm) was applied to assess the impact of the factors on IL-2 levels. The model results provided coefficients and standard errors, which we used to generate informative visualizations (Figure 5). This weighted analysis approach allowed us to obtain appropriate insights into the relationships between IL-2 measured in the supernatant of Jurkat cells and the exposure to different combinations of the simulated space stressors, while addressing the challenges posed by varying sample sizes across the different groups.

In our weighted rlm analysis (Table 2), the coefficients represent the effect of each factor on IL-2 levels measured, and the associated *t*-values indicate the significance of these effects. Significant *t*-values, marked with ‘*’, point to the factors that have a substantial impact on IL-2 expression. *t*-values greater than 1.96 or less than −1.96 were considered statistically significant at a 95% confidence level. We observe a strong effect of the addition of 1 µM HC (Stress_µM) (*t*-value = −7.0975), indicating a strong suppression of IL-2 secretion under this condition. Additionally, exposure to microgravity shows a similar significant negative effect (*t*-value = −7.4957). This aligns with the visualization of the effect in Figure 4. Moon and Mars gravity exposure have similar *t*-values, slightly lower, indicating a less strong effect on the IL-2 value. The results from the rlm model also show that for the irradiation conditions alone, the coefficient value and the significance of the effect (*t*-value) is lower.

The model shows positive coefficient signals for the combination of stress with different gravity levels, significant only for the combination of stress and microgravity. This may indicate that the effect of the combination of stress with simulated altered gravity exposure is smaller than the exposure to stress alone in normal gravity, as well as when compared to the simulated space stressors alone. However, for the vast majority of the combinations, the *t*-value is not significant or has very small significance. The same is true for the conditions representing exposure to three factors. Figure 5 provides a visual depiction of the magnitude of the coefficients with the significance values highlighting the complex interplay of factors affecting IL-2 levels.

### 2.2. IL-2 Gene Expression in Jurkat Cells after 24 h Exposure to Simulates Space Conditions

RT-qPCR was performed for the same groups, normalizing the expression of the IL-2 gene to control group expression for each experiment. The relative gene expression levels were determined using the ddCt method for quantitative PCR (qPCR), which compares the cycle threshold (Ct) values of the target gene to the reference genes and calculates the fold change in gene expression [38].

**Figure 6:**
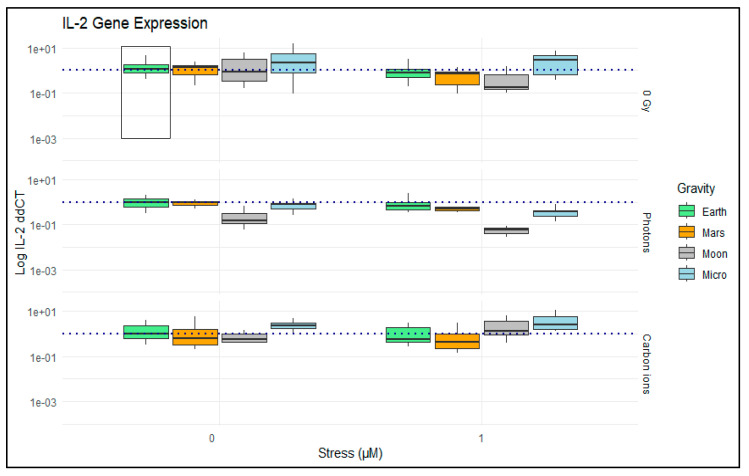
IL-2 measured on Jurkat cells using RT-qPCR. Plot shows boxplots with the median as center line. The bottom and top edges of the box represent the 1st quartile (Q1) and the 3rd quartile (Q3) of the data, respectively. The height of the box (the interquartile range or IQR) represents the middle 50% of the data. The whiskers represent 1.5 times the IQR. This figure displays data grouped by stress (µM) exposure on the *x*-axis and radiation exposure on the facet grid. The *y*-axis is represented on a logarithmic scale (log IL-2 ddCT) to better visualize a wide range of data values. The gravity factor is represented by fill colors, with light green for normal “Earth” gravity, orange for simulated Mars gravity, gray for simulated moon gravity, and light blue for simulated microgravity (“Micro”). The black line box highlights the control condition, and the horizontal dashed line indicates the average value for the control condition. N = 8 to 28. Data analyzed with RStudio v4.3.1.

We observed that the differences in the fold expression of the IL-2 gene do not change greatly across different combinations of exposure conditions (Figure 5). The effect of stress is not as evident as in the case of the IL-2 values measured in the supernatant, except for the conditions where cells are exposed to photons. In these samples, we see a decrease in the mean value of IL-2 expression across the different simulated gravity levels when hydrocortisone is added, with a more pronounced effect for the moon gravity level condition (Figure 6, gray box in stress 1 µM and photons). To note, in the simulated microgravity groups without radiation or exposure to carbon radiation, we see a tendency for increased IL-2 gene expression, independent of the addition of stress.

**Figure 7:**
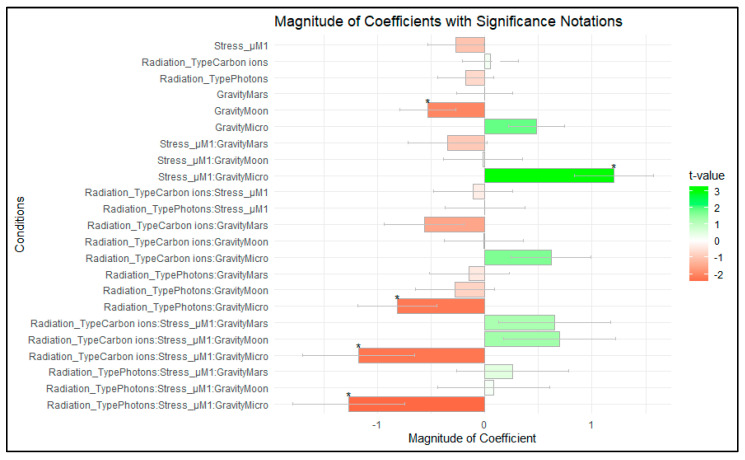
In the bar plot, *t*-values represent the significance of the coefficients for different conditions, while the coefficients’ magnitude indicates the size of the effect each condition has on the response variable. A *t*-value greater than 1.96 or less than −1.96 is considered statistically significant (marked with *). The color gradient, from red to green, illustrates both the magnitude and significance of the *t*-values. Red indicates negative *t*-values, reflecting a negative impact, while green indicates positive *t*-values, showing a positive impact. The darker the shade, the larger the magnitude of the *t*-value, emphasizing the strength of the effect. Data analyzed with RStudio v4.3.1.

qPCR data were analyzed using robust analysis to account for the lack of normality observed (see Figure S3). Outliers observed were removed. As with the ELISA results, the differences in the number of replicates per condition were accounted for by analyzing the weighted data. In the weighted rlm analysis for the RT-qPCR data (Table 3), the coefficients represent the effect of each factor on IL-2 expression, and the associated *t*-values indicate the significance of these effects. Significant *t*-values, marked with ‘*’, point to the factors that have a substantial impact on IL-2 expression. *t*-values greater than 1.96 or less than −1.96 were considered statistically significant at a 95% confidence level. Figure 7 provides an improved visualization of the results of the rlm.

### 2.3. Correlation between IL-2 Gene Expression and Supernatant Protein Levels

In order to understand if there is a decreased IL-2 gene expression that leads to the low IL-2 levels observed in the supernatant, we performed correlation analysis (Table 4).

**Figure 8:**
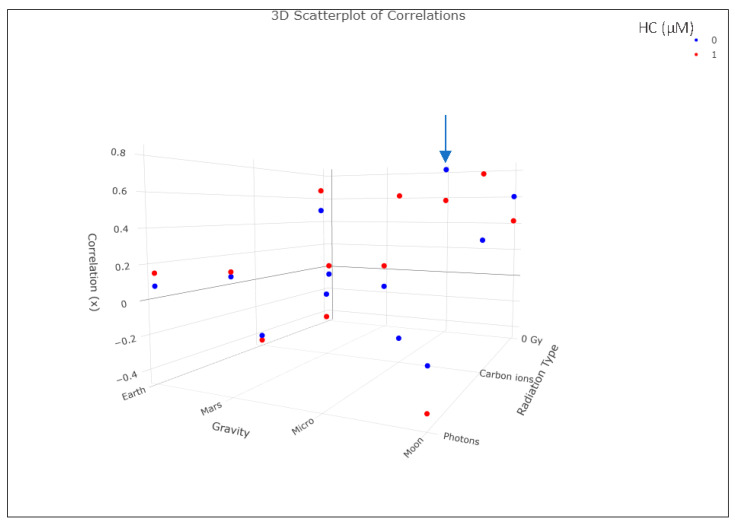
Three-dimensional scatterplot depicting the correlations (x) between IL-2 gene expression and experimental conditions. The *x*-axis represents different radiation types, with “0 Gy”, “Carbon ions”, and “Photons”. The *y*-axis illustrates variations in gravity conditions. Each data point represents a unique experimental setting corresponding to the correlation, with color indicating different stress levels (blue = 0 µM and red = 1 µM). The blue arrow signals the highest correlation value (see also Table 3). Data analyzed with RStudio v4.3.1. This is an interactive plot and can be further explored by assessing the GitHub space: https://silmarilr.github.io/Space_Immune/3D%20Scatterplot%20correlation%20data.html (accessed on 27 November 2023).

In Figure 8, we can observe a 3D rendering of the correlations between IL-2 gene expression and IL-2 supernatant protein levels. We can observe that for the microgravity condition alone, we see the closest values to one, indicating a positive correlation (also highlighted in Table 4).

**Figure 9:**
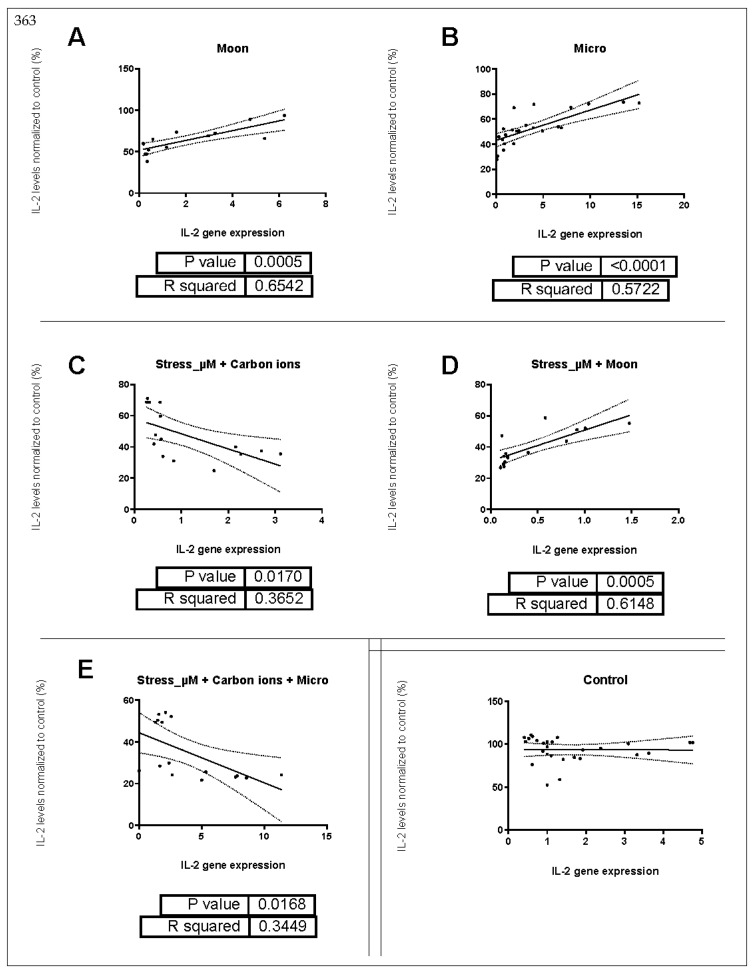
Significant simple linear regression for single exposure to simulated space conditions. Black line represents the slope of the curve, and the dotted lines are the 95% confidence intervals. Black dots represent the number of samples. (**A**,**B**) Single simulated space stressor exposure; (**C**,**D**) Double simulated space stressor exposure; (**E**) triple simulated sace stressor exposure. IL-2 expression is the ddCT value for IL-2 gene expression. P value is the result of the simple linear regression model, and R squared (R^2^) reflects the fitness of the model. Data analyzed with GraphPad Prism v10.0.3.

Simple linear regression analysis was performed to find significant correlations between IL-2 gene expression and IL-2 levels. We observe for the exposure to single simulated space stressors, that exposure to moon gravity and microgravity show a statistically significant positive relationship between IL-2 gene expression and IL-2 protein values measured in the supernatant (Figure 9A,B).

For the double exposure groups, the combination of stress and carbon ion irradiation shows a statistically significant relationship; although, the R^2^ is 0.3652, meaning that approximately 36.5% of the variability of the IL-2 levels can be explained by IL-2 gene expression, thus not accounting for the majority of the variation. (Figure 9C). Exposure to 1 µM of stress in combination with simulated moon gravity shows a positive relationship, again highlighting the correlation between IL-2 gene expression and IL-2 protein levels (Figure 9D).

Exposure to the combination of stress, carbon ion irradiation, and microgravity showed a negative relationship between IL-2 gene expression and IL-2 protein levels, again with a moderate R^2^ (Figure 9E). All the other conditions were tested, and the results can be found in Figures S5–S8.

## 3. Discussion

In this paper, we aimed to better understand the effects of different combinations of simulated space conditions on Jurkat cells, a T cell model vastly used for investigating T cell function. We hypothesized that simulated partial gravity, ionizing radiation, and the use of hydrocortisone would induce changes in Jurkat cell behavior, reflecting the impact of these simulated space conditions on T cell function. In this context, Jurkat cells were exposed to simulated microgravity, moon gravity, and Mars gravity, photons and carbon ions, as well as HC. Furthermore, we aimed to investigate the impact of these factors alone or in different combinations in order to understand their individual impact. To achieve these goals, we conducted a series of experiments and assessed the expressed and secreted IL-2 protein values in cell culture supernatant and the IL-2 gene expression levels in Jurkat cells. The results of our study demonstrated significant changes in Jurkat cell behavior under different combinations of simulated space conditions.

Expressed and secreted IL-2 protein concentration measured in the supernatant showed that exposure to ionizing radiation, simulated altered gravity, and stress diminished IL-2 amounts secreted by the cells in different magnitudes. Moreover, the combination of simulated space further altered the cells’ response. This suggests a suppressive effect on T cell function, as IL-2 plays a crucial role in T cell activation, which seems to align with other works. In this context, previous work on Jurkat cells and peripheral blood mononuclear cells (PBMCs) obtained from astronauts showed reduced capacity to produce IL-2 following space flight or microgravity exposure [39,40]. In addition, whole blood cultures from both space shuttle and ISS astronauts showed as well that T cells when activated, had a decreased production of IL-2 [41].

The effects of a combined exposure to (simulated) space stressors is understudied. Hence, in real flight experiments, it becomes fairly difficult to account for the influence of each of them. Recent publications aimed to tackle the effects of radiation and microgravity, showing an additive effect on osteoclast fusion [10]. Also, skin cells are affected by spaceflight. Fibroblast function under different combinations of simulated space stressors was assessed by Radstake et al. Results show interactions between different spaceflight stressors affecting different cellular processes [42]. With regard to the immune system, several in vitro studies using different cell types have been focusing on the effects of simulated microgravity on the DNA damage response (DDR) to ionizing radiation [43,44], which can be translated into a higher risk for astronauts. Murine models were also used to investigate the effects of combined simulated microgravity (hind limb unloading model (HLU)) and proton irradiation, showing decreased activation and proliferation indices of splenic T lymphocytes after combining proton irradiation and HLU [45].

Partial gravity exposure is a relatively novel technology available for simulating Mars and moon gravity levels. In a systematic review assessing the current knowledge on partial gravity exposure and the response of biomechanical and cardiopulmonary systems, the authors highlight that partial gravity levels are not sufficient to maintain optimal physiological functions [13]. The effects on immune system responses to this altered gravity level are understudied, and the need to bridge the gap related to the effects to different gravity levels (from hypergravity to microgravity) is highlighted by what we have observed in the response of the Jurkat cells to Mars gravity exposure, where a more pronounced decrease was observed when compared to moon gravity exposure. Mechanosensing mechanisms might be at play in the context of the observed effect of T cell activation translated by IL-2 secretion suppression. An investigation conducted by M. ElGindi et al. looked at how a 3D microenvironment can ameliorate the effect of simulated microgravity on activated Jurkat cells [46].

In this paper, we have shown that hydrocortisone exposure alone leads to decreased IL-2 levels measured in stimulated Jurkat cells’ supernatant, indicating a decrease in their pro-inflammatory capacity. Glucocorticoids are known to regulate immune activity and can act as Th1 cell suppressors while promoting Th2 activity, which causes an overall reduced pro-inflammatory activity [47]. Exposure to altered levels of gravity showed a decrease in the IL-2 levels measured, which decreased further when it was combined with hydrocortisone exposure. Ionizing radiation on the other hand, showed very little effect on IL-2 levels. However, when this exposure was combined with the addition of hydrocortisone, we observed a substantial decrease in IL-2 secretion levels. These results seem to indicate that hydrocortisone can influence the sensitivity of Jurkat cells to ionizing radiation. Radstake et al. also showed that the addition of cortisol to fibroblasts altered their response to simulated microgravity and radiation exposure [42]. Chronic stress has also been suggested to have implications on the outcome of radiotherapy treatments through the suppression of the immune system [48].

Effects of exposure to ionizing radiation vary greatly with the quality of the radiation used. As mentioned in Section 1, the complexity of the space radiation field derives, in part, from differences in the LET of the mixed field. To understand how T cells respond to different radiation qualities, we exposed our cells to photons (X-rays) and carbon ions. We can observe a bigger impact of X-ray irradiation on IL-2 levels when compared to carbon ions alone. One explanation might be related to the activation of the nuclear factor κB (NF-κB). Hellweg et al. have shown that high LET irradiation leads to a stronger activation of the NF-κB pathway, when compared to X-rays, in a response that might be correlated with DNA damage complexity [49,50]. For the immune system, NF-κB is a pivotal pathway for the induction of inflammation with IL-2 as one of the outcomes of its activation. This might explain why exposure to carbon ions has less of a negative impact on the IL-2 secreted protein levels. However, the observed changes are altered when the exposure is combined with simulated altered gravity, as we can see a larger decrease in IL-2 levels with lower gravity levels for carbon ion exposures and not for the X-ray-irradiated groups. This might highlight the importance of the effect of altered gravity (particularly microgravity) on the DDR response, thus affecting IL-2 production through NF-κB activation [31].

### Limitations and Future Perspectives

Jurkat cells have been a T cell model that has been, and still is, fairly commonly used across several studies on T cell activation and function [51]. However, Jurkat cells are lymphoblastic in nature, thereby having a cancerous background, which should be taken into account. For example, the metabolic changes that are characteristic of cancers cells can interfere with their activation capacity and response to stimuli [52]. Also *TP53*, *BAX* genes are known to be mutated in these cells, and, in the context of radiation exposure, DDR responses could be abnormal [53]. Future work should focus on the use of normal T cells, for example, donor derived, that might better reflect the effects of simulated space stressors on the immune system.

In our paper, we used mitogenic activators for the activation of Jurkat cells, which work independently of the T cell receptor (TCR). This broad activation strategy might be different on a molecular level than fine-tuned TCR activation, which characterizes the more elegant immune system responses in the human body. Future works should aim to use specific activators of T cells, such as the TCR activators anti-CD3/anti-CD28.

In order to properly simulate altered gravity conditions with a minimal amount of shear stress, we devised a system where our cells were in airtight vials. However, this results in a lack of oxygenation and CO_2_ buffering of the cell culture media. As cell toxicity was a concern, we limited our exposure time to 24 h. However, this time limit resulted in other considerations. Associated with the limited 24h experimental design, a higher dose of hydrocortisone was used, which was proven to show observable effects. Some studies have shown that astronauts have experienced levels of plasma cortisol of up to 0.610 µmol/L during spaceflight [54]. This high concentration of cortisol surpasses the threshold proposed by the inverted U model, where concentrations of this hormone above ~0.45 µgl/dL (~1µmol/L) can be considered detrimental [55]. In the future, different hydrocortisone concentrations can be explored in order to establish a threshold for the alterations observed at 1 µM. This could prove beneficial as the astronauts’ hydrocortisone levels could be monitored during long-duration space flight missions.

In our paper, the IL-2 gene expression data do not fully reflect the alterations observed in the secreted IL-2 protein levels. In this context, Thiel et al. showed that T cells responded to gravity alterations as soon as 20 s after start of the exposure by changing their gene expression [30]. The transcription rate for the IL-2 gene has been shown to increase in the initial phase of activation but decrease steadily even in the presence of the activators. Concrete degradation of mRNA occurs around 12–16 h, while transcription continues to be active [56]. Other time-points for assessing gene expression kinetics will shed better light on the complex response of T cells to simulated space stressors. Moreover, IL-2 may have a negative feedback role in the activation of T cells. Data provided by microarray have shown that the cytokine can regulate the mRNA levels of known signaling regulators that inhibit downstream components of the pathway initiated by T cell receptors [57]. This can also lead to a decreased IL-2-mediated activation and secretion, independent of mRNA levels. Together, this could explain why there is no correlation between IL-2 mRNA and protein levels in our experiments.

In the future, deep space missions, for example, towards Mars, will see astronauts ionizing radiation exposures reach ~1 Sv. For X-rays, this is equivalent to 1 Gy and for GCR exposures, depending on the outcome and tissue used as reference, it is approximately 0.4 Gy [58]. Currently, the isoeffective dose for higher LET irradiations of immune cells is not known; therefore, we opted to use the same deposition of energy (1 Gy) for both irradiation types. Although the contribution of carbon ions present in the GCR spectra to the radiobiological effects is not as prominent as the one from heavier ions (like iron for example), the high LET achieved in this setup, and a comparison that was made with photon irradiation, allowed us to explore LET-specific differences in the response of the Jurkat cells. Future investigations should aim to use more space-relevant ions or protons, or, ideally, GCR simulators such as the one available at the NASA space radiation laboratory [59] or at GSI Helmholtzzentrum für Schwerionenforschung [60]. In this work, ionizing radiation exposure was achieved with two different experimental setups due to the nature of the radiation beam lines. While the total dose applied was 1 Gy at a dose rate of approximately 0.5 Gy/minute, the constancy of the deposition of carbon ions was less stable than that of photon irradiation, influencing the dose rate. Moreover, as mentioned, in space, the accumulated dose on a Mars round trip (approximately 500 days) would be approximately 1 Sv, with relatively low dose rates (1.84 mSv/day) [61]. However, in our setup, cells cannot be kept in culture for more than 24 h; therefore, an acute irradiation needed to be administered. Future works should consider lower dose rates that better match dose rates found in space and can be achieved in ground-based facilities, using, for example, in vivo models [62]. Additionally, irradiation of the samples with carbon ions, although the distribution of the particles is optimized there, might also have an influence on the response when compared to the uniform field obtained during photon irradiation. Other dosimetric considerations are the use of air-KERMA for the calculation of the photon dose, whereas for carbon ion irradiation, absorbed dose in water was used, which could lead to moderate differences in terms of the dose administered to the cells. This should be taken into account in future irradiations.

IL-2 is a key molecule in the orchestrated immune response; however, other cytokines are also important, especially considering pro-inflammatory vs. inflammatory responses. Jurkat cells produce high amounts of IL-2 upon stimulation, but previous work conducted in our lab showed that other important cytokines such as interferon-γ (IFN- γ) or IL-10 are only found in very low concentrations in the supernatant, which would make it difficult to analyze their concentrations in the context of exposure to simulated space conditions (values could be below the detection limit). Future works utilizing this setup should aim to use other cellular T cell models and perform analysis on a full panel of cytokines, both at the protein and the transcriptomic level, in combination with other functional assays.

Another important factor to consider is the use of in vitro models to study the response of the immune system to simulated space stressors. The immune response is complex and orchestrated by several cells, tissues, and organs. The use of a single type of immune cell does not fully reflect the complex relationships between different cells required for an immunological response. In this context, the use of isolated PBMCs and exposing them to simulated space conditions could help elucidate the more complex relationships between different immune cells in the human peripheral blood. Another option is the use of co-culture systems in which the interaction between immune cells and other cell types could be explored.

With future space missions aiming to go longer and further into our solar system, maintaining a healthy immune system is crucial to help protect astronauts in order to achieve successful missions. In this paper, we show that single exposure to different radiation qualities, simulated altered gravity, or HC decrease IL-2 concentration of stimulated Jurkat cells, significantly for HC, Mars, and microgravity. Separation of the exposure to different simulated space stressors can help us better understand which component of the space environment causes an effect on the human body, which in turn, will help space agencies make more informed decisions on the use of countermeasures, such as shielding technologies or stress management protocol implementations.

## 4. Materials and Methods

### 4.1. Cell Culture

Jurkat cell line clone E6-1 was used. Cells were obtained from American Type Culture Collection (ATCC^©^, Manassas, VA, United States of America (USA)) and handled according to the provider specifications. The base medium for this cell line is ATCC-formulated Roswell Park Memorial Institute (RPMI) 1640 Medium (Gibco™), supplemented with 10%, heat-inactivated Fetal Bovine Serum (FBS) (Gibco™), 1x Minimum Essential Medium Non-Essential Amino Acids (GIBCO^®^), 1% Penicillin-Streptomycin (10,000 U/mL, Gibco™), and 50 µM of 2-Mercaptoethanol solution (1115 g/mL, Sigma-Aldrich^®^, Saint Louis, MO, USA). Jurkat cells were kept in incubator at 37 °C with 5% CO_2_ in air atmosphere. Subculturing was performed with the addition of fresh medium every 2–3 days and cell density was kept at ~1.0 × 10^6^/mL for all experiments.

### 4.2. Irradiation

Photon irradiation was performed at the Laboratory of Nuclear Calibration of the Belgian Nuclear Research Centre (SCK CEN) in Mol, Belgium. Cell culture flasks were placed on a Plexiglass plate in a horizontal position and irradiated from the top at a 50 cm distance and irradiated with X-rays (Irradiator Xstrahl 320 kV H-250, air kerma (Kair) 1.00 Gy with uncertainty on conventional value (k = 2) of 0.06 Gy). Carbon ion irradiation was performed at the *Grand Accelerateur de Ions de Lourdes* (GANIL), in Caen, France. Fluoro-ethyl polymer (FEP) cell culture bags (PL07-2G, Origen Biomedical, Austin, TX, USA) were used for irradiation of the Jurkat cells in order to obtain an even distribution of the dose (due to the requisite that the samples are placed vertical to the beam line window and Jurkat cells are suspension cells). In both irradiations, cells were irradiated with a total dose of 1 Gy.

### 4.3. ivDTH—Stimulation Assay

Activation of the Jurkat cells was achieved with the addition of a cocktail of substances that induce a strong production of IL-2. Phytohemagglutinin-L (PHA-L, eBioscience™, San Diego, CA, USA, 00-4977-03) and Phorbol Myristate Acetate (PMA, P1585, Sigma-Aldrich^®^, Saint Louis, MO, USA) were used in combination with Ionomycin (I3909, Sigma-Aldrich^®^, Saint Louis, MO, USA) at a final concentration in the cell suspension of 1 μg/mL PHA, 50 ng/mL PMA, 1 μg/mL Ionomycin, [63,64,65]. Shortly before use, stock solutions from each component of the cocktail were prepared in Dimethylsulfoxide (DMSO, 5.89569, Sigma-Aldrich^®^, Saint Louis, MO, USA). For the unstimulated control conditions, vehicle DMSO was used. According to the experimental group (see Figure 3), 1 × 10^6^ cells were transferred to cryogenic vials which were used for the experimental setup. Each vial consisted of a biological replicate, and 15 vials were used per condition for each experimental campaign.

### 4.4. Simulated Altered Gravity

Simulated microgravity, moon gravity, and Mars gravity conditions were created using an RPM 1.0 and 2.0 (Yuri Gravity). In order to prevent the occurrence of air bubbles in the cell suspension, the vial caps were sealed using a polymer (SYLGARD 184 Silicone Elastomer, Dow, 01673921), allowing for airtight sealing of the vial. To account for the lack of gas exchange during the experimental setup time, the media were previously allowed to reach gas equilibrium in the incubator (37 °C with a 5% CO_2_ in air atmosphere). Cells were exposed to simulated altered gravity for 24 h, and any replicate vials with air bubbles were discarded.

### 4.5. Hydrocortisone

Hydrocortisone (HC), a synthetic hormone similar to cortisol, is commonly used in medical treatments for its anti-inflammatory properties. A stock solution of 1 mg/mL (2.76 mM) of HC (Sigma-Aldrich^®^, H0888) was made in DMSO. This stock solution was further dissolved in DMSO to obtain a concentration of 100 µM. The HC solution was 1/100 diluted in cell culture media to obtain a final working HC concentration of 1 µM. To the conditions not exposed to HC, DMSO vehicle was added. The cells were exposed to HC for 24 h.

### 4.6. ELISA

IL-2 levels were measured in the supernatant of the Jurkat cells after the 24 h ivDTH. Cells were spun down at 1000 g for 8 min, and the supernatant was collected and kept at −80 °C until used. The Thermo Fisher Scientific human IL-2 Uncoated ELISA Kit was used according to the manufacturer’s protocol. Samples were diluted 1/50 for accurate calculations based on the standard curve for each plate. Plates were measured using the Absorbance 96 plate reader by Byonoy at 450/570 nm. The output was further analyzed using Belysa^®^ Immunoassay Curve Fitting Software (Merck, Rahway, NJ, USA).

### 4.7. Real-Time qPCR

RNA was extracted from the cell pellet obtained after supernatant collection. The pellet was lysed with RLT plus buffer (Qiagen, Hilden, Germany) with 1:100 β-mercaptoethanol. RNA was isolated using the RNeasy Plus Mini Kit (Qiagen) following the manufacture’s protocol. Purity and concentration of the isolated RNA were verified with the NanoDrop™ 2000/2000 (Thermo Scientific™, Waltham, MA, USA). For each sample, RNA was reverse-transcribed into cDNA using the GoScript™ Reverse Transcriptase (Promega, Alexandria, Australia).

The cDNA was used with a dilution of 5 ng/μL for all samples. IL-2 qPCR primer pair was obtained from Sino Biological (Sino Biological, Inc., Beijing, China). Housekeeping genes primers were custom designed and purchased from Eurogentec (Kaneka Eurogentec, Seraing, Belgium). The sequences are shown in Supplementary Table S3. All real-time PCR reactions were performed using the qTOWER^3^ Series (Analytik Jena GmbH + Co., Jena, Germany) using the SYBR Green PCR Master Mix (Applied Biosystems, Waltham, MA, USA). The thermal cycling conditions were 60 °C for 2 min followed by an initial denaturation step at 95 °C for 10 min, 45 cycles at 95 °C for 30s, 60 °C for 30 s, and 72 °C for 30 s. The relative quantification in gene expression was determined using the 2 ^−ΔΔCt^ method [66].

### 4.8. Data Analysis

Data visualization and statistical analysis were obtained using RStudio version 4.3.1 or GraphPad Prism version 10.0.3. In order to estimate the required sample size, a power analysis was performed in a simulation setting. Assuming that the knowledge about the population comes from the collected dataset, a linear model with triple interaction, including stress, radiation type, and gravity, was applied. The power was calculated for different values of the sample size. Finally, the sample size for which the null hypothesis was rejected in 80% of cases was selected for the analysis, corresponding to a power of 80%. The calculations accounted for a possibly slightly higher uncertainty. Additional explanation on statistical analyses can be found in the Supplementary Files SM1 and SM2.

## 5. Conclusions

To the best of our knowledge, this is the first study on Jurkat cells that uses different combinations of simulated space stressors in order to investigate their effect on T cell function. Moreover, this is the first study using simulated Mars and moon gravity levels on Jurkat cells in order to assess the immune function differences between different gravity levels.

We have shown that different simulated space stressors by themselves have different impacts on the IL-2 levels expressed and secreted by Jurkat cells. The combination of simulated space stressors may have different impacts on IL-2 protein levels, with HC being a factor in the reduction of IL-2 concentration across combinations. IL-2 gene expression is altered during exposure to simulated space conditions, but understanding the kinetics of this response is important for future work.

Further investigations that aim to understand the mechanisms behind these changes are crucial for the development of effective countermeasures to account for the prevalent space-induced immune dysfunction experienced by astronauts. Psychological stress and possible alterations of basal hydrocortisone levels combined with ionizing radiation and altered gravity exposure should be studied in the context of the immune system to decrease the health risks during future deep space exploration missions.

## Figures and Tables

**Table 1:** Listing of the different conditions in the experimental setup.

Radiation Exposure	Concentration of HC (Stress)	Gravity
0 Gy	0 µM	Earth 1 g
1 Gy X-rays (photons)	1 µM	Mars~0.38 g
1 Gy carbon ions		Moon~0.17 g
		Micro~0.01 g

**Table 2:** Results from the robust linear model for the IL-2 levels.

	Coefficient Value	Std. Error	*t* Value	
Carbon ions	−7.7845	5.3472	−1.4558	
Photons	−15.7117	5.3472	−2.9383	*
Stress µM	−37.9513	5.3472	−7.0975	*
Mars	−36.0043	5.3472	−6.7333	*
Moon	−28.9804	5.3472	−5.4198	*
Micro	−40.4247	5.3931	−7.4957	*
Mars—Stress µM	8.2061	7.562	1.0852	
Moon—Stress µM	13.6749	7.562	1.8084	
Micro—Stress µM	17.86	7.5946	2.3517	*
Carbon ions—Stress µM	−0.2715	7.6116	−0.0357	
Photons—Stress µM	−0.4461	7.562	−0.059	
Carbon ions—Mars	2.742	7.6144	0.3601	
Carbon ions—Moon	−16.7909	7.6663	−2.1902	*
Carbon ions—Micro	−3.0143	7.6439	−0.3943	
Photons—Mars	−11.3448	7.562	−1.5002	
Photons—Moon	24.4772	7.562	3.2368	*
Photons—Micro	37.5884	7.5946	4.9494	*
Carbon ion—Mars—Stress µM	13.8434	10.7664	1.2858	
Carbon ions—Moon—Stress µM	13.3951	10.8032	1.2399	
Carbon ions—Micro—Stress µM	11.9049	10.7873	1.1036	
Photons—Mars—Stress µM	26.3041	10.6943	2.4596	*
Photons—Moon—Stress µM	15.231	10.6943	1.4242	
Photons—Micro—Stress µM	9.4366	10.7173	0.8805	

— Expresses combination of the simulated space stressors; * indicates *t*-values greater than 1.96 or less than −1.96.

**Table 3:** Results from the robust linear model for the IL-2 gene expression.

	Coefficient Value	Std. Error	*t* Value	
Carbon ions	0.0511	0.2608	0.1958	
Photons	−0.1743	0.2608	−0.6682	
Stress µM	−0.2668	0.2608	−1.023	
Mars	0.002	0.2608	0.0077	
Moon	−0.5274	0.2608	−2.0222	*
Micro	0.4839	0.2608	1.8556	
Mars—Stress µM	−0.3441	0.3688	−0.9329	
Moon—Stress µM	−0.0157	0.3688	−0.0426	
Micro—Stress µM	1.2016	0.3688	3.2579	*
Carbon ions—Stress µM	−0.1111	0.3688	−0.3011	
Photons—Stress µM	0.0027	0.3688	0.0072	
Carbon ions—Mars	−0.5645	0.3688	−1.5304	
Carbon ions—Moon	−0.0096	0.3688	−0.026	
Carbon ions—Micro	0.617	0.3688	1.6727	
Photons—Mars	−0.1429	0.3688	−0.3876	
Photons—Moon	−0.2765	0.3688	−0.7496	
Photons—Micro	−0.8147	0.3688	−2.2089	*
Carbon ions—Mars—Stress µM	0.6473	0.5216	1.241	
Carbon ions—Moon—Stress µM	0.6935	0.5216	1.3295	
Carbon ions—Micro—Stress µM	−1.1722	0.5216	−2.2473	*
Photons—Mars—Stress µM	0.2568	0.5216	0.4923	
Photons—Moon—Stress µM	0.0872	0.5216	0.1671	
Photons—Micro—Stress µM	−1.2668	0.5216	−2.4287	*

— Expresses combination of the simulated space stressors; * indicates *t*-values greater than 1.96 or less than −1.96.

**Table 4:** Correlation between IL-2 gene expression and supernatant protein levels.

Radiation_Type	Stress_µM	Gravity	Correlation
0 Gy	0	Earth	−0.06304529
Carbon ions	0	Earth	−0.40766668
Photons	0	Earth	0.07761725
0 Gy	0	Mars	−0.1410919
Carbon ions	0	Mars	−0.0699926
Photons	0	Mars	0.15788835
0 Gy	0	Micro	→0.81937225
Carbon ions	0	Micro	−0.32210807
Photons	0	Micro	0.49734382
0 Gy	0	Moon	0.60214769
Carbon ions	0	Moon	0.322216
Photons	0	Moon	−0.18010503
0 Gy	1	Earth	0.01094268
Carbon ions	1	Earth	−0.44159913
Photons	1	Earth	0.15052512
0 Gy	1	Mars	0.03330089
Carbon ions	1	Mars	−0.22493537
Photons	1	Mars	0.18267156
0 Gy	1	Micro	0.57702244
Carbon ions	1	Micro	0.58951685
Photons	1	Micro	0.589298
0 Gy	1	Moon	0.4198696
Carbon ions	1	Moon	0.71189189
Photons	1	Moon	−0.39601785

→ highlights the closest value to 1.

## Data Availability

All data are available upon request.

## Supplementary Materials

The following supporting information can be downloaded at: https://www.mdpi.com/article/10.3390/ijms242316943/s1.
