# Supplementary material for: Lost in Space? Unmasking the T Cell Reaction to Simulated Space Stressors
Source: Int J Mol Sci. 2023 Nov 29;24(23):16943. doi: 10.3390/ijms242316943 (PMC10707245; doi:10.3390/ijms242316943)
Supplement: Supplementary file 1 [file ijms-24-16943-s001.zip › ijms-2718348-supplementary.pdf]

SUPPLEMENTARY MATERIALS (SM) TO

Lost in Space? Unmasking the T-cell Reaction to Space Stressors

Silvana Miranda[1,2], Randy Vermeesen[1], Whilemina Radstake[1,2], Alessio Parisi[3, 4] Anna Ivanova[5], Sarah Baatout[1,2], Kevin Tabury[1,2], and Bjorn Baselet [2,*]

SM1 Normality testing for the ELISA dataset and fitness of the robust linear model

Table S1. Summary of the data frame for the IL-2 levels

Radiation_Type	Stress_µM	Gravity		IL_2_levels	
Length: 460	Length: 460	Earth	140	Min.	11.32
Class :character	Class :character	Mars	120	1st Qu.	36.93
Mode :character	Mode :character	Moon	80	Median	55.95
		Micro	120	Mean	59.77
				3rd Qu.	77.04
				Max.	153.21
				NA's	5

Normality testing after outlier removal

Normality tests were performed, specifically the Shapiro-Wilk and Anderson-Darling tests. The results indicated that our data significantly deviates from a normal distribution, with p-values of 1.345e-09 and 2.571e-11, respectively. This suggests that the assumption of normality may not hold. Furthermore, the skewness value was found to be 0.6149376, indicating a slight rightward skew, while the kurtosis was -0.148114, indicating a slightly flatter distribution than the normal distribution. These findings suggest that our data may not meet the normality assumption for parametric statistical tests.

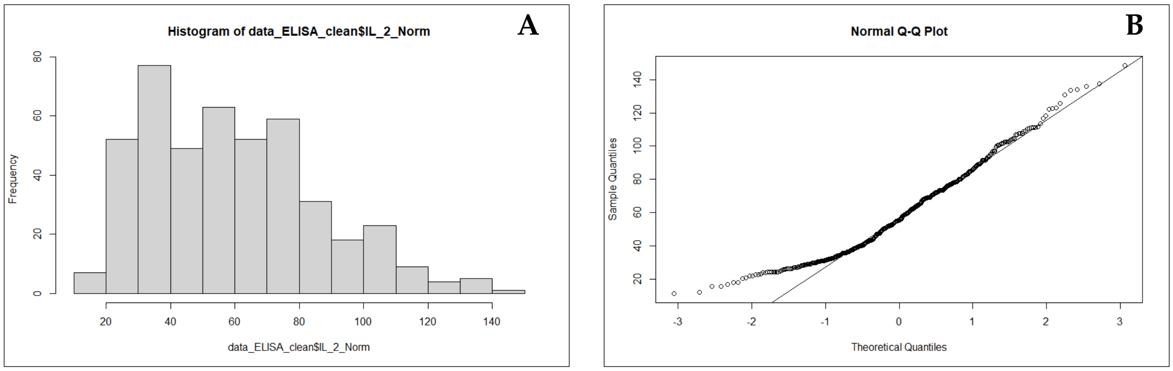

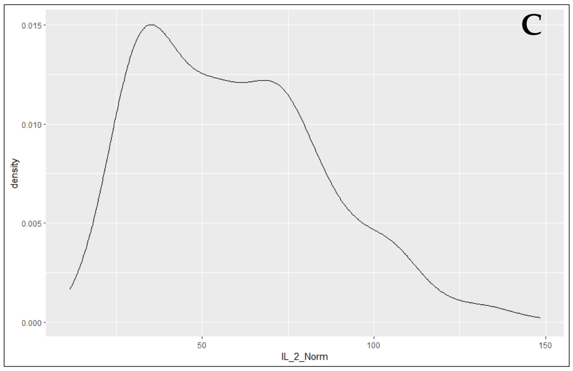

Figure S1. Visual plots for assessing the normality of the data. A: histogram of the distribution ; B: Normal Quantile-Quantile (Q-Q) plot; C: Density plot

Normality tests were also performed in the residuals of linear regression model fitted. The p-value from the Shapiro-Wilk Test is 5.837e-07, which is much smaller than the typical significance level of 0.05. This indicates strong evidence against the null hypothesis that the residuals are normally distributed. Also the p-value is 6.35e-07 from the Anderson-Darling test is significantly smaller than 0.05, suggesting that the residuals deviate from normality. Kolmogorov-Smirnov Test resulted in the p-value < 2.2e-16, indicating a significant departure from normality.

This tests reinforced the need to apply robust linear regression models.

Fitness of the robust model

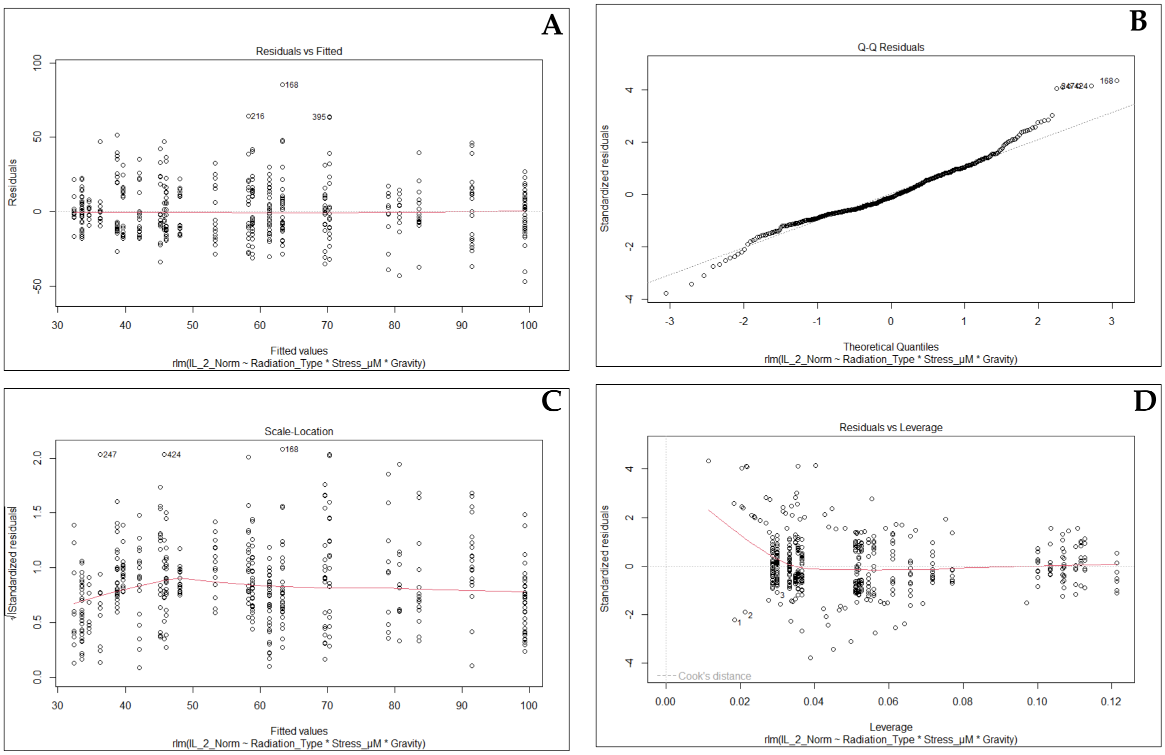

Figure S2. Visual inspection of the fitness of the robust model. A: Residuals vs fitted values (heteroscedasticity verification); B: Q-Q of the residuals. C: Scale-Location plot (homoscedasticity verification); D: Leverage-Residual plot.

SM2 Normality testing for the qPCR dataset and fitness of the robust linear model

Table S2. Summary of the data frame for the IL-2 expression

Radiation_Type	Stress_µM	Gravity		IL_2_levels	
Length: 351	Length: 351	Earth	108	Min.	0.000138
Class: character	Class: character	Mars	90	1st Qu.	0.427177
Mode: character	Mode: character	Moon	61	Median	0.878746
		Micro	92	Mean	1.579442
				3rd Qu.	1.939064
				Max.	15.200677

Normality testing after outlier removal

Normality tests were also performed for the qPCR data. The Shapiro-Wilk and Anderson-Darling tests results indicated again a significant deviation from a normal distribution, with p-values of < 2.2e-16 for both tests. In this case, the skewness value was found to be 1.544965, indicating a long right sided tail. The kurtosis was 2.110967, suggesting that the data has heavier tails than a normal distribution. These findings suggest that our qPCR does not meet the normality assumption for parametric statistical tests.

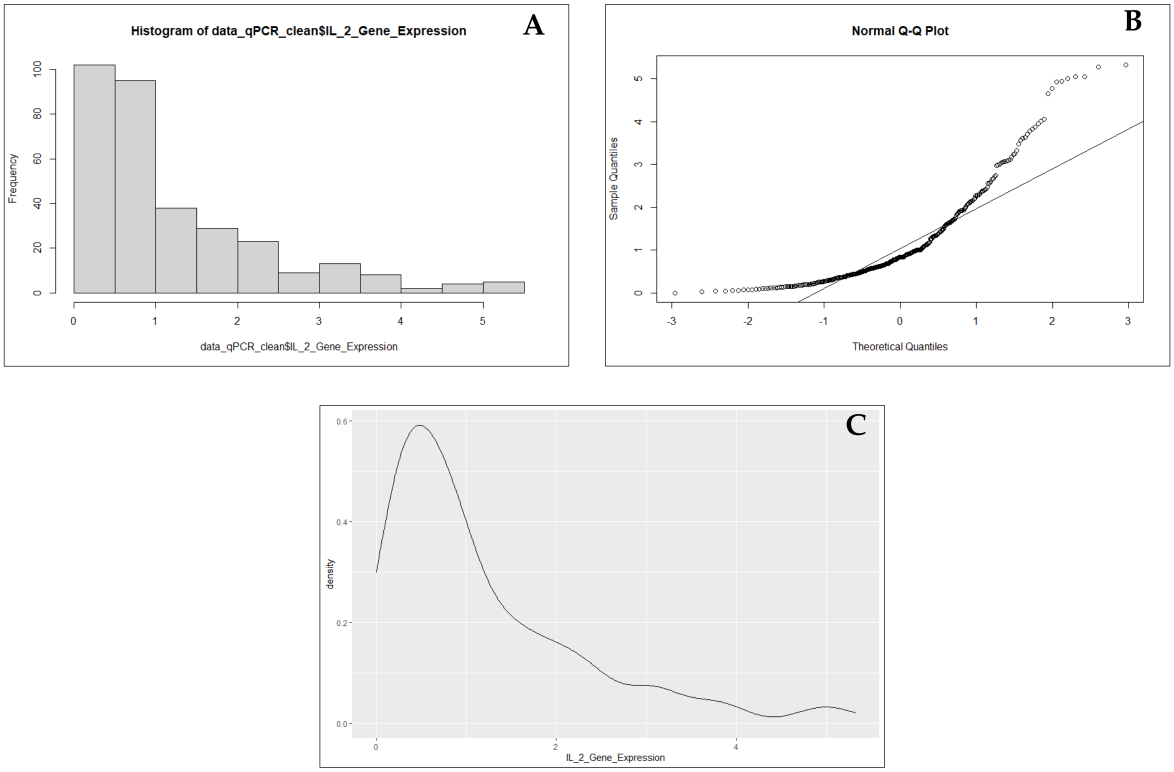

Figure S3. . Visual plots for assessing the normality of the data. A: histogram of the distribution ; B: Normal QQ plot; C: Density plot

Fitness of the robust model

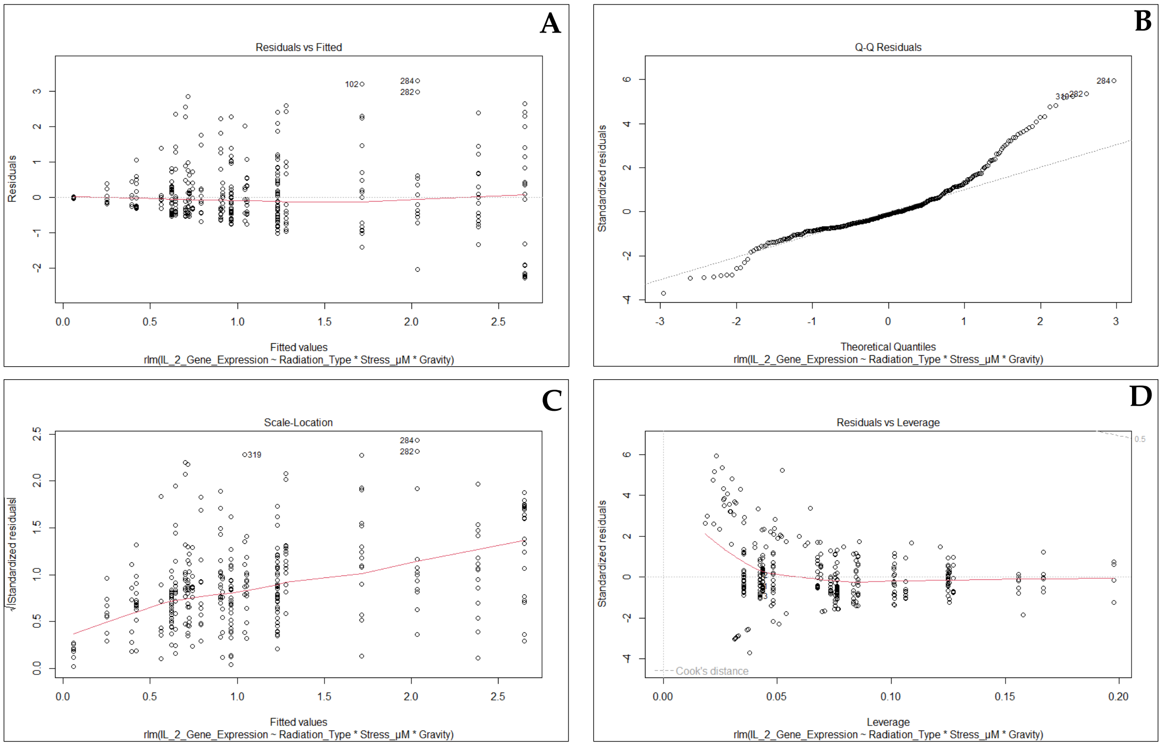

Figure S4. Visual inspection of the fitness of the robust model. A: Residuals vs fitted values (heteroscedasticity verification); B: Q-Q of the residuals. C: Scale-Location plot (homoscedasticity verification); D: Leverage-Residual plot

SM3 Additional simple linear regression graphs

Simple linear regression was performed for each group.

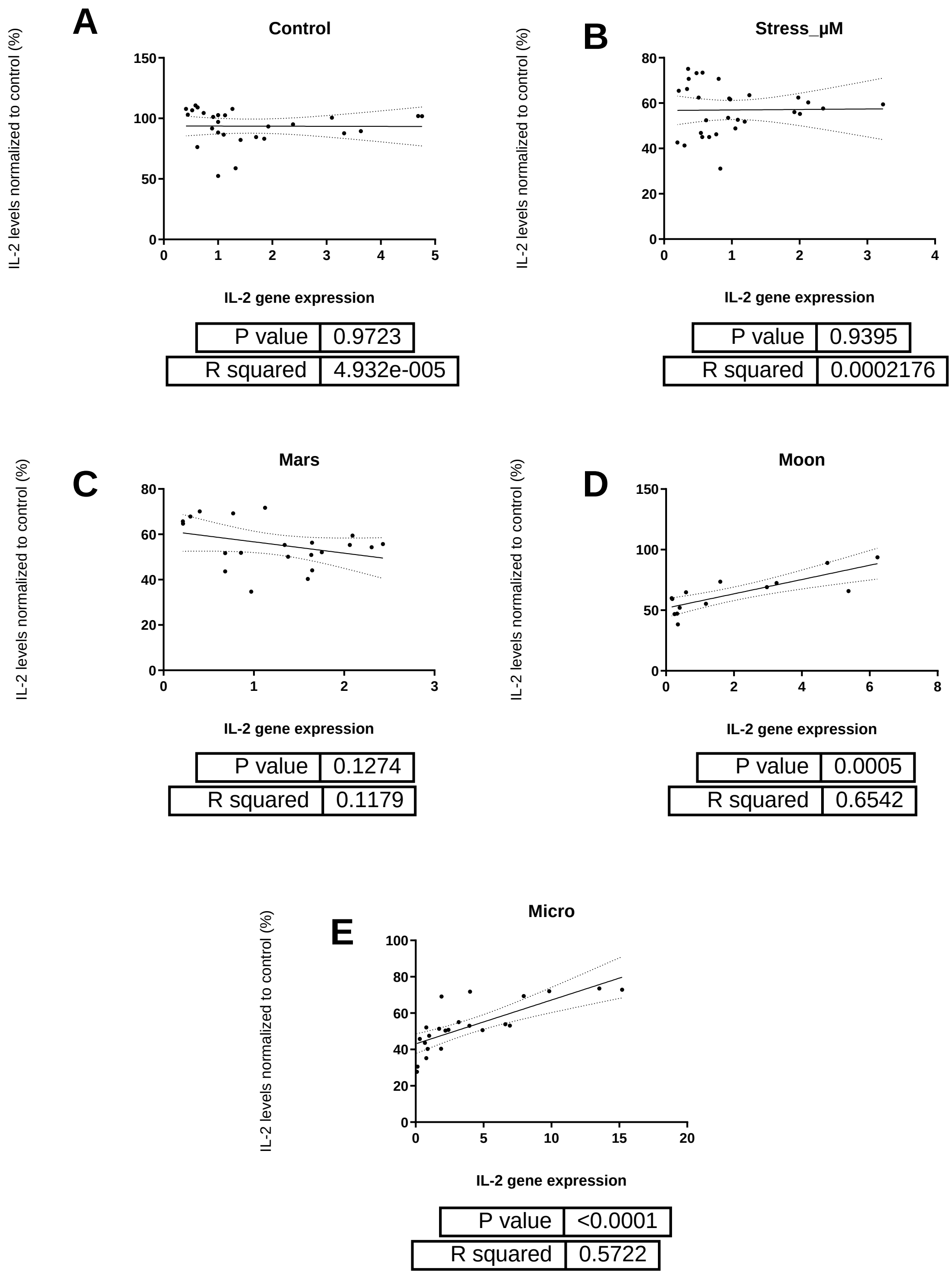

Figure S5. Simple linear regression for single stressor exposures

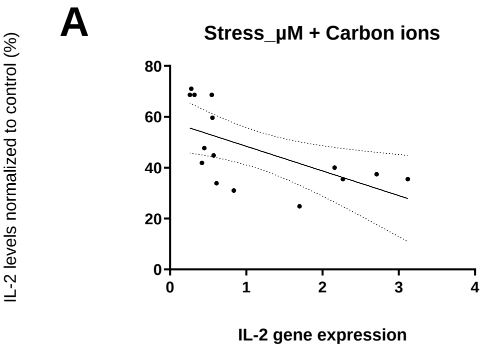

P value	0.0170
R squared	0.3652

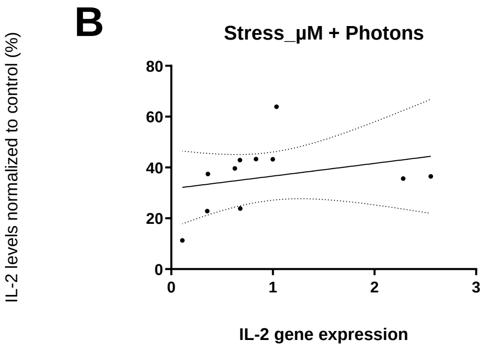

P value	0.3981
R squared	0.08042

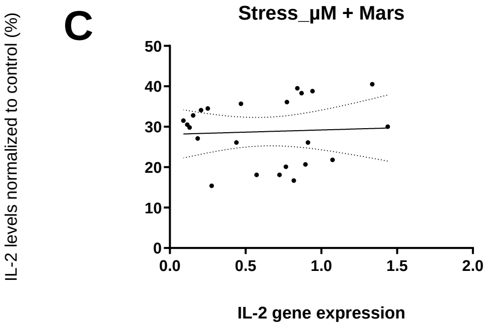

P value	0.8049
R squared	0.002972

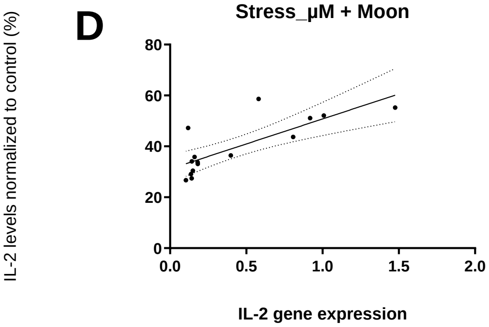

P value	0.0005
R squared	0.6148

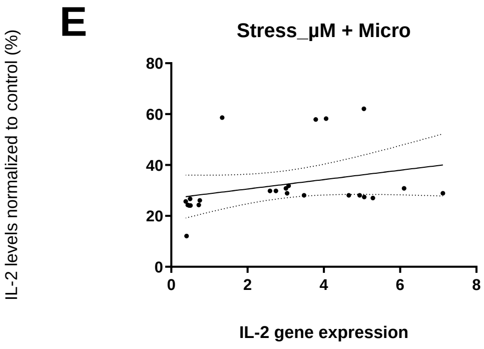

P value	0.1557
R squared	0.08944

Figure S6. Simple linear regression for stress in combination with other simulates space conditions

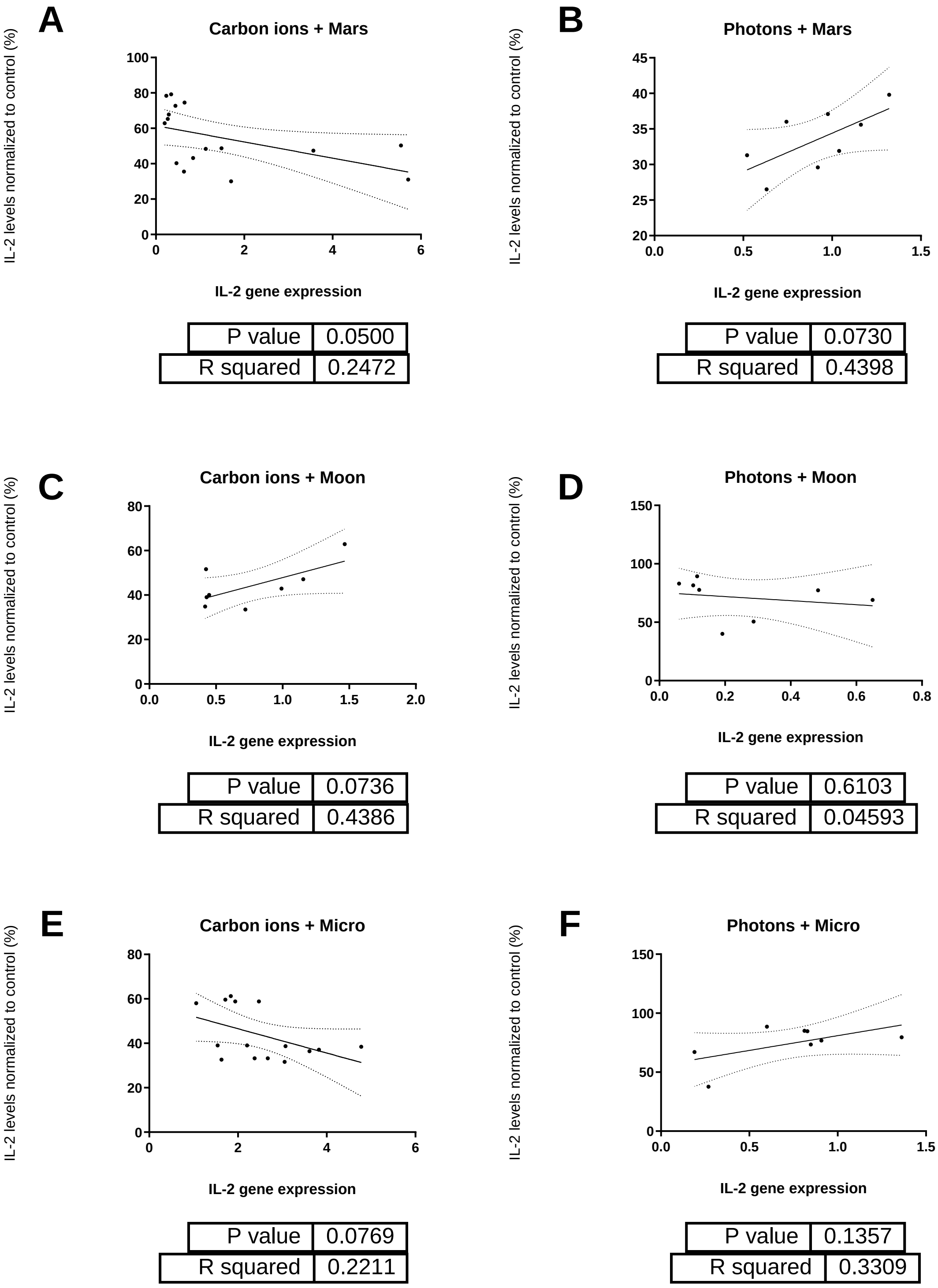

Figure S7. Simple linear regression for combing different radiation qualities with different simulated gravity levels

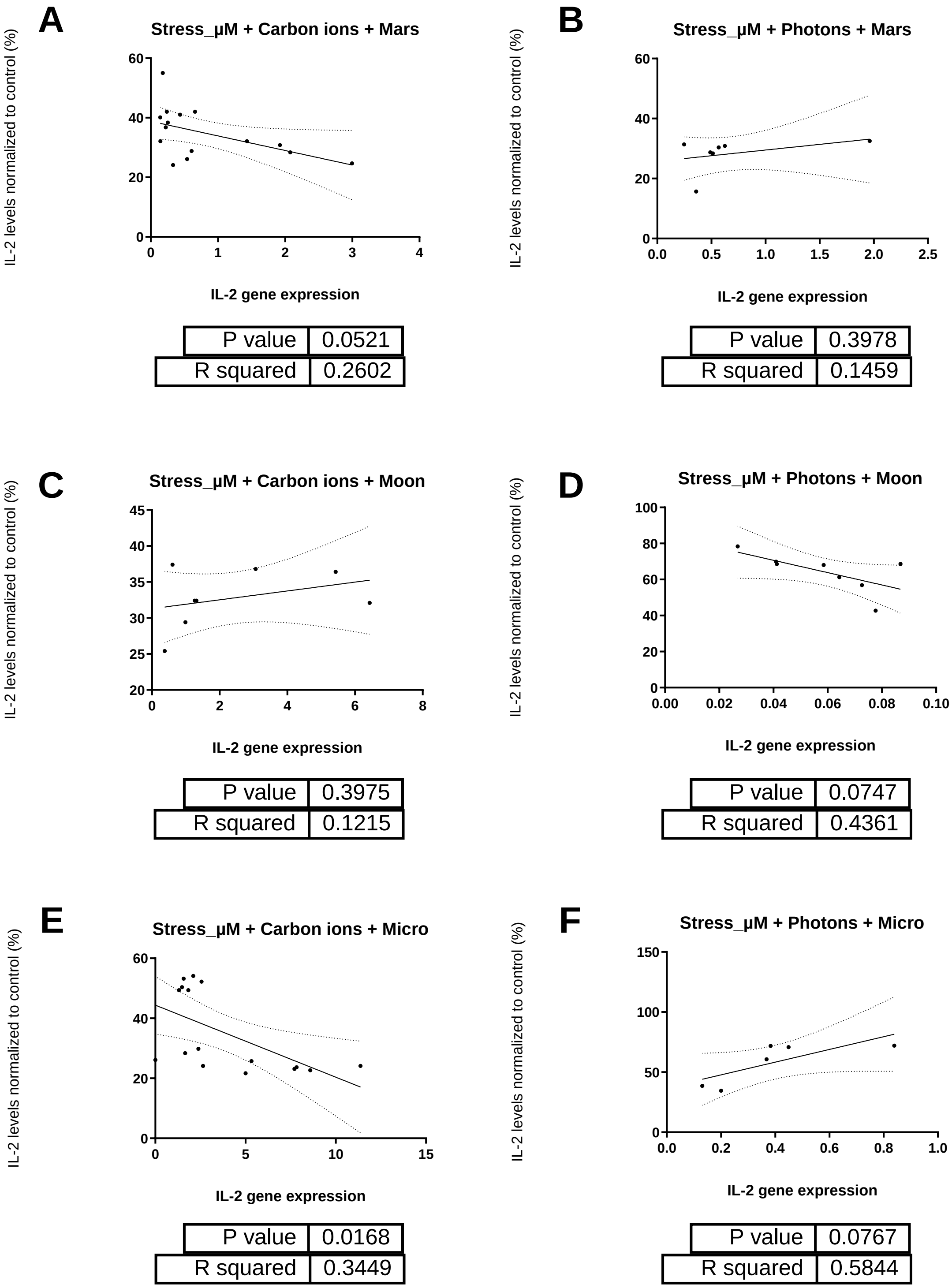

Figure S8. Triple exposure to simulated space stressors

SM4 Housekeeping genes sequences

Table S3. Forward and reverse sequences of housekeeping genes used in RT-qPCR

Gene	Forward Sequence	Reverse Sequence
18s rRNA	GTAACCCGTTGAACCCCATT	CCATCCAATCGGTAGTAGCG
HPRT	CATTATGCTGAGGATTTGGAAAGG	CTTGAGCACACAGAGGGCTACA
